# The Association between Two-Stage Tourniquet Application during Total Knee Replacement and Blood Loss: A Retrospective Cohort Study

**DOI:** 10.3390/jcm11061682

**Published:** 2022-03-18

**Authors:** Min Seok Oh, Ji-Yoon Kim, Cho Long Kim, Su Rim Koh, Yundo Jung, Na Yeon Kim, Mi Ae Jeong

**Affiliations:** 1 Department of Anesthesiology and Pain Medicine, College of Medicine, Hanyang University, Seoul 04763, Korea; oms21st@hanyang.ac.kr; 2 Department of Anesthesiology and Pain Medicine, Hanyang University Hospital, Seoul 04763, Korea; irisjy00@hanmail.net (J.-Y.K.); starkcl@naver.com (C.L.K.); rhtnfla@gmail.com (S.R.K.); fcfrankfurty@gmail.com (Y.J.); kny951103@naver.com (N.Y.K.)

**Keywords:** total knee replacement, arthroplasty, tourniquet, transfusion, vital stability

## Abstract

Tourniquet use during total knee arthroplasty improves the surgical field, but is associated with several complications. The medical records of 506 patients who underwent elective total knee arthroplasty or total knee replacement from January 2017 to December 2020 were reviewed. A total of 331 patients who had undergone total knee arthroplasty were included. In the first half course group, the tourniquet was inflated with a pressure of 300 mmHg after manual banding before the incision and deflated after cement insertion. In the two-stage group, the tourniquet was inflated and deflated at the same stages of the procedure as in the first half course group. However, in this second group, the tourniquet was deflated for 15 min and then inflated again, and, finally, it was deflated after skin closure. The estimated blood loss, the number of patients who needed medications to control their blood pressure, and opioid usage at the post-anesthesia care unit were similar in both groups. The two-stage tourniquet technique was not related to reduced total blood loss in total knee arthroplasty.

## 1. Introduction

With a growing population of older adults in Korea, the number of knee arthroplasty procedures is increasing annually. The number of joint arthroplasty operations increased from 64,515 in 2010 to 85,592 in 2020. Tourniquet use during total knee arthroplasty (TKA) or total knee replacement (TKR) improves the surgical field of view [1,2] and facilitates cement injection. Additionally, it has the advantage of reducing the amount of bleeding [3] during and after surgery, and shortening operation times [4]. However, it can sometimes cause damage to nerves [5], blood vessels, and muscles, causing swelling or restrictions to the postoperative range of motion [6,7]. Several studies have demonstrated that pain and swelling after surgery can be reduced by reducing the tourniquet application time or lowering the tourniquet pressure [8], but this is still a controversial topic [2]. The typical duration of tourniquet application in TKA is from the beginning to the end of the procedure [9]. However, this tends to destabilize the patient’s vital signs [10,11,12] and increase the amount of fluid or blood administered. Sudden restoration of blood flow after long-term tourniquet application may impair the circulation of blood to the cardiovascular system or cerebrovascular system, thereby worsening the patient’s prognosis [13]. Reducing the tourniquet time or lowering the pressure may disturb the surgeon’s field of view, increase the operation time, or reduce the accuracy of the operation [14]. Recently, during TKA at our hospital, we have been implementing a two-stage application process, involving tourniquet re-application after 15 min of tourniquet off-time after cement injection. In the past, the tourniquet was only applied until the injection of cement, but we switched to the method of applying the tourniquet again after a 15 min tourniquet off-time, until skin closure. As the duration of uninterrupted tourniquet inflation increased the likelihood of neural dysfunction [15], it was expected that this method would reduce the amount of bleeding by applying the tourniquet until the end of skin closure, but would not increase the complications due to the 15 min resting period. In previous studies, the outcomes have been compared with a lack of tourniquet use [6], loosening the tourniquet after cement injection, or even after the skin incision [16,17]. However, to the best of our knowledge, no published studies have investigated the risks and benefits associated with tourniquet reapplication after an intra-operative rest period. This study analyzed differences in estimated blood volume loss, blood transfusion requirements, medications during and after surgery, and analgesic usage in the recovery room between patients who underwent TKA with tourniquet application until cement insertion (during the first 2 years in which we used this protocol) and patients who underwent TKA with two-stage tourniquet application (during the final 2 years in which our hospital used this protocol).

## 2. Materials and Methods

### 2.1. Patients

This retrospective, single-center cohort study was approved by Hanyang University Seoul Hospital’s Institutional Review Board (HYUH 2021-08-041-003), which waived the requirement for written informed consent. The medical records of 506 patients who underwent elective TKA from January 2017 to December 2020 were assessed for eligibility, and 414 patients were enrolled. Eligible patients underwent unilateral TKA for the first time or contralateral TKA during one hospitalization. The exclusion criteria were revision operation, surgery on both legs at one time, and surgery under spinal anesthesia. All patients included in the study were operated on by a single senior orthopedic surgeon during this period.

The following two groups were compared according to the duration of tourniquet application: the first half (FH) course group versus the two-stage (TS) group. In the FH group, the tourniquet was inflated with a pressure of 300 mmHg after manual banding before the incision, and was deflated after cement insertion. After cement fixation, bleeding control, and muscle and skin closure were started. In the TS group, tourniquet inflation began and deflated at the same stage of the procedure as in the FH group. However, the tourniquet was deflated for 15 min (if the cement was fixed within 15 min, bleeding control was started) and then inflated again during muscle and skin closure; the tourniquet was deflated after skin closure.

### 2.2. Perioperative Anesthetic Care

After entering the operating room, all patients were monitored for blood pressure, heart rate, oxygen saturation, and anesthesia depth. Anesthesia induction was performed with 1–1.5 mg/kg propofol, along with 0.1 μg/kg/min remifentanil and sevoflurane. Anesthesia was maintained with inhalational anesthetic gas and remifentanil. Mechanical ventilation was delivered at a tidal volume of 6–8 mL/kg using a mixture of oxygen and medical air at a flow rate of 2–3 L/min. Arterial blood pressure was monitored via the right or left radial artery to evaluate the hemoglobin level. A 16 G large-bore angiocatheter was placed in the external jugular vein to infuse fluid and blood products. The target perioperative systolic arterial pressure was 80 to 160 mmHg, and, if necessary, cardiovascular agents, such as calcium channel blockers, beta-blockers, ephedrine, and phenylephrine, were used. After induction and during muscle closure, hemoglobin levels were checked via arterial blood analysis. Tranexamic acid was not used. If hemoglobin was less than 8 g/dL, packed red blood cells (RBCs) were transfused. One unit of packed RBCs was approximately 320 mL in volume, of which the red blood cell volume was 180 to 200 mL. Hemovac drain was placed under the skin at the end of the surgery.

### 2.3. Outcome Variables

The following variables were further evaluated: (1) pre-operative factors (age, sex, weight, height, and comorbidities); (2) intra-operative factors (perioperative hemoglobin level, tourniquet time, and use of cardiovascular agents); (3) post-operative factors (post-operative hemoglobin level, transfusion of packed RBCs, and use of analgesics).

### 2.4. Statistical Analysis

All statistical analyses were performed using SPSS Statistics for Windows, version 27 (IBM Corp., Armonk, NY, USA). Categorical variables are expressed as numbers and percentages. Continuous variables are reported as means ± standard deviations. Normally distributed data were evaluated with the Shapiro–Wilk test or the Kolmogorov–Smirnov test.

Primary outcomes (hemoglobin and estimated blood loss) were evaluated with the Mann–Whitney U test or independent *t*-test.

Demographic data, peri-operative data, and clinical outcomes between the two groups were analyzed using the chi-square test for categorical variables, and an independent samples *t*-test or Mann–Whitney U test for continuous variables. For skewed data, the Mann–Whitney U test was used. Differences in categorical variables were compared by the chi-square test or Fisher’s exact test. A two-sided alpha of 0.05 was used for all statistical tests.

## 3. Results

**Figure 1:**
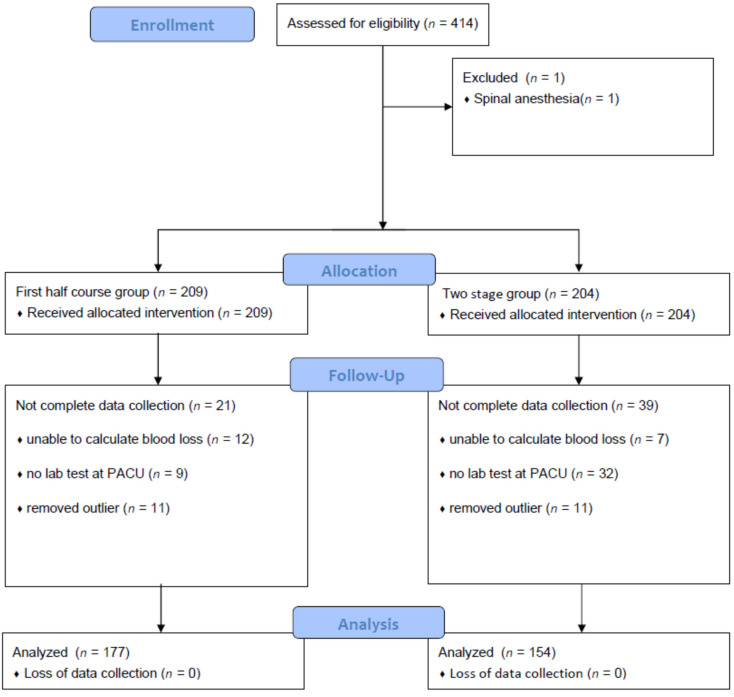
Flow diagram of patient selection.

A total of 414 cases of unilateral TKA surgery were reviewed. Among the 414 patients, one patient who underwent spinal anesthesia was excluded. There were 209 patients in the FH group and 204 patients in the TS group. In each group, we excluded 12 and 7 patients, respectively, for whom it was impossible to calculate the amount of bleeding, and 9 and 32 patients, respectively, with missing hemoglobin levels because laboratory tests were not performed in the recovery room immediately after surgery. We also excluded 11 patients from each group with outlying values of blood volume loss. Finally, data were analyzed for 331 operations (Figure 1).

There were no significant differences in age, height, or weight between the groups. There was no significant intergroup difference resulting from the independent samples *t*-test analysis. Cardiovascular diseases, such as hypertension, were more prevalent in the TS group. There were no significant differences between the groups in terms of patients who stopped taking anticoagulants that could affect the bleeding volume (Table 1). There was no significant intergroup difference in pre-operative hemoglobin level. 

Intra-operative blood loss was calculated using the hemoglobin balance formula [3,18]. The FH group had a mean blood loss of 542.90 mL, and the TS group had a mean bleeding volume of 514.66 mL (Table 2).

$$\begin{array}{l} \begin{array}{l} \mathrm{Hbloss}\;\mathrm{total}=\mathrm{BV}\;\times \;\left(\mathrm{Hbi}\;-\;\mathrm{Hbe}\right)\;\times \;0.001+\mathrm{Hbt}.\\ \mathrm{Vloss}\;\mathrm{total}=1000\;\times \;\mathrm{Hbloss}\;\mathrm{total}/\mathrm{Hbi}.\\ \mathrm{Generally},\;1\;U\;\mathrm{banked}\;\mathrm{blood}\;\mathrm{is}\;\mathrm{considered}\;\mathrm{to}\;\mathrm{contain}\;52\;\pm \;5.4\;g\;\mathrm{Hb}.\\ \mathrm{BV}=k1\;\times \;H3+k2\;\times \;W+k3.\\ \mathrm{For}\;\mathrm{males},\;k1=0.3669,\;k2=0.03219,\;\mathrm{and}\;k3=0.6041; \end{array}\\ \mathrm{For}\;\mathrm{females},\;k1=0.3561,\;k2=0.03308,\;\mathrm{and}\;k3=0.1833. \end{array}$$

Vloss total (mL): the total volume of RBC loss;

Hbloss total (g): the loss volume of Hb;

Hbi (g/L): the Hb value before surgery;

Hbe (g/L): the Hb value after surgery;

Hbt (g): the total volume of blood transfusion.

There was no significant difference between the two groups in the proportions of patients who used antihypertensive drugs while using tourniquets, or those who used antihypertensive drugs after tourniquet removal (Table 3).

Intra-operative transfusions were lower in the TS group. There was no statistically significant intergroup difference in transfusion volume in the recovery room, or in transfusion requirement in the ward after the surgery (Table 4). However, although not statistically significant, the transfusion volume in the ward was generally lower in the TS group than in the FH group.

The hemoglobin values were compared intra-operatively and in the post-anesthesia care unit. The hemoglobin values were similar in both groups (Table 5).

Since the estimated blood loss and hemoglobin values do not differ significantly between the two groups, an analysis of the intergroup differences was performed on data for transfused patients and non-transfused patients (Table 6).

Pre-operative hemoglobin and initial intra-operative hemoglobin were significantly lower in transfused patients. Lower hemoglobin values were related to intra-operative transfusion.

The total opioid usage was compared in terms of fentanyl dose (Table 7). Pethidine 25 mg was converted to fentanyl 25 µg equivalents. The analgesic demand was relatively larger in the TS group, but the intergroup difference was not statistically significant.

There was no significant difference between the ischemic time from the first tourniquet in the TS group versus the total tourniquet time in the FH group (Table 8).

## 4. Discussion

Intra-operative tourniquet use has been studied extensively. Tourniquets are used in most operations because it is thought that they help secure the field of view and shorten operation times accordingly. However, it is known that skin blistering, wound hematoma, wound oozing, muscle injury, rhabdomyolysis, nerve palsy, postoperative stiffness, deep vein thrombosis, and pulmonary embolism [6] may be associated with tourniquet use. At our hospital, we investigated associations between the method of tourniquet use in TKA operations and blood loss, need for medication, and opioid consumption.

The reason we first introduced this method was that events such as a gradual decrease in the patient’s blood pressure sometimes occurred at the time of suture. Therefore, we assumed that if the tourniquet was applied again, the amount of bleeding at the time of muscle and skin suturing could be reduced, even if only a little.

In a previous study [16], a significantly reduced bleeding volume was associated with prolonged tourniquet application. In our study, there was no significant difference in the amount of intra-operative bleeding between the FH and TS groups, despite a slightly lower amount of blood loss in the TS group. Significantly, lower levels of intra-operative bleeding have been associated with tourniquet application compared with when tourniquets are not used [6]. In previous studies, the mean blood loss volumes when using tourniquets have varied from 25.6 mL to 350 mL. In our study, the estimated mean blood loss was 542.90 mL in the FH group, compared with 514.66 mL in the TS group.

Blood loss estimates vary greatly from study to study because of differences between studies in the formulas used to calculate the amount of bleeding. According to one study [18], the different mean values obtained ranged from 971 mL to 1699 mL, depending on which of the four formulas was used to calculate the bleeding volume. In this study, the amount of bleeding was estimated using the hemoglobin balance formula.

As for transfusion requirements, the proportion of patients who underwent intra-operative transfusion was statistically significantly higher in the FH group. There was no statistically significant intergroup difference in post-operative blood transfusion requirements in the post-anesthesia care unit or ward, but, on average, they were lower in the TS group. As can be observed from the results, the pre-operative hemoglobin and initial intra-operative hemoglobin were significantly lower in transfused patients. The reason that the proportion of patients who needed transfusion was higher in the FH group might be related to the incidence of patients who had lower hemoglobin levels. Although additional research is needed to determine what levels of pre-operative hemoglobin increase the possibility of transfusion, if the patient’s hemoglobin is not high, preparing packed red blood cells in advance might be a better option.

In a comparative study of tourniquet application versus non-application, the amount of bleeding during surgery was small in the tourniquet group, but the amount of bleeding after surgery showed mixed results [19]. However, if blood transfusions could be reduced, even during surgery, this might help to reduce the complications associated with blood transfusions, such as urticaria, anaphylaxis, transfusion-related acute lung injury, and hypothermia [20].

In this study, the total operation time was around 130 min. In previous studies [19], the operation time varies greatly from 73 to 163 min.

Prolonged tourniquet use increases the patient’s blood pressure and pulse rate, which, in turn, causes severe hypotension after turning the tourniquets off. It is known that the relative mortality risk increases by 3.6% for every minute of hypotension, which is defined as an SBP of less than 80 mmHg [21]. Despite the fact that about 20 min of tourniquet time was added, in this study, the need for anti-hypertensive drugs, such as nicardipine or beta-blockers, to lower the blood pressure during tourniquet use, or the need for vasopressors, such as ephedrine or phenylephrine, after tourniquet release, were not significantly different between the two groups. The rest period in the middle is thought to be helpful for preventing hemodynamic insults that blunt the sympathetic activity [22] of tourniquet use throughout an entire operation. As this was a retrospective study, the blood pressure and heart rate at the exact time before and after tourniquet removal were not recorded. Usually, the anesthetic record is completed every 5 min, so the exact time of vital signs could not be determined.

In addition, there was no significant intergroup difference in the amount of analgesic used in the recovery room after surgery, so it is thought that there was no significant difference in the pain felt by the patients. The relationship between the initial visual analog scale score and subsequent opioid requirement is depicted by a sigmoid curve [23].

Through this retrospective study, we could not find a significant correlation between the method of tourniquet use and the amount of bleeding in TKA. However, it is widely thought that tourniquet use is associated with small volumes of blood transfusion.

There were some limitations to this study.

First, this study was not a randomized controlled trial. However, there was no statistically significant difference in age or body mass index between the two patient groups, and there was no significant difference between the two groups in the number of patients who used anticoagulants that could affect the amount of bleeding.

Second, since this was a retrospective study, it was not possible to set standards for the use of vasopressors or transfusions during or after surgery. However, the groups did not significantly differ in this regard.

A well-designed randomized controlled trial is needed to further investigate the two-stage application of tourniquets.

## 5. Conclusions

The two-stage tourniquet technique was not related to reduced total blood loss in total knee replacement.

## Figures and Tables

**Table 1:** Characteristics of patients.

Variables	FH Group	TS Group	*p*-Value
	(*n* = 177)	(*n* = 154)	
Patient Characteristics			
Age	69.5 ± 8.3	69.9 ± 7.2	0.622
Height (cm)	155.1 ± 6.4	154.9 ± 7.7	0.756
Weight (kg)	62.0 ± 9.7	62.1 ± 9.2	0.899
Disease characteristics			
Hypertension	97	103	0.025
Diabetes Mellitus	39	35	0.880
Stroke	6	1	0.084
Chronic kidney disease	6	6	0.806
Angina, myocardial infarction	7	13	0.087
Taking anticoagulants	42	45	0.258
Pre-operative hemoglobin (g/dL)	12.9 ± 1.2	13.0 ± 1.1	0.589

**Table 2:** Estimated blood loss by hemoglobin balance formula.

Estimated Blood Loss	FH Group	TS Group
Volume (mL)	542.90 ± 274.77	514.66 ± 228.54

*p* = 0.314.

**Table 3:** The number of patients who needed medications.

Medications	FH Group (*n* = 177)	TS Group (*n* = 154)	*p*-Value
Type of Drugs			
Antihypertensive drug ^1^	38	26	0.292
Vasopressor ^2^	28	21	0.577

^1^ nicardipine, labetalol, and esmolol; ^2^ phenylephrine and ephedrine.

**Table 4:** Transfused blood units during and after the surgery.

Transfused Blood (Number of Patients)	FH Group	TS Group	*p*-Value
Intra-operatively	0(149)	0(151)	0.000
	1(26)	1(3)	
	2(2)	2(0)	
In the post-anesthesia care unit	0(174)	0(153)	0.385
	1(3)	1(1)	
In the ward post-operatively	0(153)	0(140)	0.108
	1(12)	1(11)	
	2(12)	2(3)	

**Table 5:** Hemoglobin values during and after the surgery.

Hemoglobin Values (g/dL)	FH Group	TS Group	*p*-Value
Intra-operatively (initial)	11.8 ± 1.1	11.8 ± 1.0	0.435
Intra-operatively (last)	10.5 ± 1.0	10.4 ± 1.0	0.403
In the post-anesthesia care unit	11.3 ± 1.0	11.2 ± 1.0	0.246

**Table 6:** Hemoglobin values of transfused and non-transfused patients.

Hemoglobin Values (g/dL)	Non-Transfused Group (*n* = 300)	Transfused Group (*n* = 31)	*p*-Value
Pre-operative	13.1 ± 1.0	11.7 ± 1.0	0.000
Intra-operatively (initial)	11.9 ± 1.0	10.5 ± 0.8	0.000

**Table 7:** Opioid usage at post-anesthesia care unit.

Opioid Use (µg)	FH Group	TS Group
Fentanyl	76.2 ± 43.9	84.4 ± 56.1
Equivalent		

*p* = 0.147.

**Table 8:** Durations of tourniquet applications.

Time (min)	FH Group (*n* = 177)	TS Group (*n* = 154)	*p*-Value
Tourniquet applied			
1st	89.0 ± 17.2	89.6 ± 16.6	0.747
2nd	-	25.4 ± 8.2	

## Data Availability

The data presented in this study are available on request from the corresponding author.
